# Stimulation of Autophagy by Dapagliflozin Mitigates Cadmium-Induced Testicular Dysfunction in Rats: The Role of AMPK/mTOR and SIRT1/Nrf2/HO-1 Pathways

**DOI:** 10.3390/ph16071006

**Published:** 2023-07-14

**Authors:** Hany H. Arab, Ebtehal Mohammad Fikry, Shuruq E. Alsufyani, Ahmed M. Ashour, Azza A. K. El-Sheikh, Hany W. Darwish, Abdullah M. Al-Hossaini, Muhammed A. Saad, Muhammad Y. Al-Shorbagy, Ahmed H. Eid

**Affiliations:** 1 Department of Pharmacology and Toxicology, College of Pharmacy, Taif University, P.O. Box 11099, Taif 21944, Saudi Arabia or hany.arab@pharma.cu.edu.eg (H.H.A.); s.alsofyani@tu.edu.sa (S.E.A.); 2 Department of Biochemistry, Faculty of Pharmacy, Cairo University, Cairo 11562, Egypt; 3 Department of Pharmacology, Egyptian Drug Authority (EDA)—Formerly NODCAR, Giza 12654, Egypt; ebtehal21@yahoo.com (E.M.F.); drahmedhamdy2007@yahoo.com (A.H.E.); 4 Department of Pharmacology and Toxicology, College of Pharmacy, Umm Al Qura University, P.O. Box 13578, Makkah 21955, Saudi Arabia; amashour@uqu.edu.sa; 5 Basic Health Sciences Department, College of Medicine, Princess Nourah bint Abdulrahman University, P.O. Box 84428, Riyadh 11671, Saudi Arabia; aaelsheikh@pnu.edu.sa; 6 Department of Pharmaceutical Chemistry, College of Pharmacy, King Saud University, P.O. Box 2457, Riyadh 11451, Saudi Arabia; hdarwish@ksu.edu.sa (H.W.D.); abalhossaini@ksu.edu.sa (A.M.A.-H.); 7 Department of Pharmaceutical Sciences, College of Pharmacy, Gulf Medical University, Ajman 4184, United Arab Emirates; dr.shorbagy@gmu.ac.ae; 8 Department of Pharmacology and Toxicology, Faculty of Pharmacy, Cairo University, Cairo 11562, Egypt

**Keywords:** dapagliflozin, cadmium, testicular impairment, autophagy, apoptosis, oxidative stress

## Abstract

Cadmium (Cd) is a widespread environmental pollutant that triggers testicular dysfunction. Dapagliflozin is a selective sodium-glucose co-transporter-2 inhibitor with notable antioxidant and anti-apoptotic features. It has shown marked cardio-, reno-, hepato-, and neuroprotective effects. Yet, its effect on Cd-evoked testicular impairment has not been examined. Hence, the goal of the current study was to investigate the potential positive effect of dapagliflozin against Cd-induced testicular dysfunction in rats, with an emphasis on autophagy, apoptosis, and oxidative insult. Dapagliflozin (1 mg/kg/day) was given by oral gavage, and testicular dysfunction, impaired spermatogenesis, and biomolecular events were studied via immunohistochemistry, histopathology, and ELISA. The current findings demonstrated that dapagliflozin improved relative testicular weight, serum testosterone, and sperm count/motility and reduced sperm abnormalities, signifying mitigation of testicular impairment and spermatogenesis disruption. Moreover, dapagliflozin attenuated Cd-induced histological abnormalities and preserved testicular structure. The testicular function recovery was prompted by stimulating the cytoprotective SIRT1/Nrf2/HO-1 axis, lowering the testicular oxidative changes, and augmenting cellular antioxidants. As regards apoptosis, dapagliflozin counteracted the apoptotic machinery by downregulating the pro-apoptotic signals together with Bcl-2 upregulation. Meanwhile, dapagliflozin reactivated the impaired autophagy, as seen by a lowered accumulation of SQSTM-1/p62 and Beclin 1 upregulation. In the same context, the testicular AMPK/mTOR pathway was stimulated as evidenced by the increased p-AMPK (Ser487)/total AMPK ratio alongside the lowered p-mTOR (Ser2448)/total mTOR ratio. Together, the favorable mitigation of Cd-induced testicular impairment/disrupted spermatogenesis was driven by the antioxidant, anti-apoptotic, and pro-autophagic actions of dapagliflozin. Thus, it could serve as a tool for the management of Cd-evoked testicular dysfunction.

## 1. Introduction

Exposure to harmful environmental toxicants has been demonstrated to exert a negative impact on the reproductive system, resulting in infertility [1,2]. As a widely utilized heavy metal in the industrial and agricultural fields, cadmium (Cd) poses an unavoidable exposure danger to human health [2]. In this regard, Cd is found in electrical conductors, heating elements, tires, electronic components, photocells, and polyvinyl chloride plastic products (PVC) [3]. Moreover, cigarette smoking and phosphate fertilizers have been regarded as significant sources of cadmium exposure [1].

Due to the rapid absorption of Cd and its long half-life in humans, it prompts detrimental health effects even at low doses [4,5]. In the context of reproductive toxicity, Cd has been reported to possess gonadotoxic and spermiotoxic properties, likely via acting as an endocrine disruptor [3,4]. In acute and chronic studies, Cd exposure has been shown to elicit a deleterious impact on the function and structure of male reproductive organs. In this context, Cd leads to a number of abnormalities, such as Sertoli cell dysfunction, testicular steroidogenesis abrogation, serum testosterone reduction, germ cell death, sperm motility/quality reduction, and prostate cancer [1,2,3]. At the cellular and molecular levels, the harmful impact of Cd is brought about when Cd crosses the blood–testis barrier, which causes an increase in oxidative events and apoptosis of germ cells [1,5,6]. With respect to oxidative perturbations, the vulnerability of testicular tissue to reactive oxygen species (ROS) attack is due to its increased content of testicular polyunsaturated fatty acids. These noxious events instigate testicular dysfunction, diminished sperm motility, and ultimately lead to infertility [5,6]. In testicular tissues of Cd-intoxicated rodents, inhibition of the silent information-regulated transcription factor 1 (SIRT1)/nuclear factor erythroid 2-related factor-2 (Nrf2)/heme oxygenase-1 (HO-1) cascade has been reported. This event has been characterized by diminished protein expression of SIRT1 together with its downstream antioxidant signals, Nrf2 and HO-1 [7], favoring the occurrence of testicular damage [7,8]. Moreover, the pathology of Cd-evoked testicular disruption reveals that apoptotic cell death is crucial for testicular damage. The apoptotic events are marked by the downregulation of the anti-apoptotic B cell lymphoma-2 protein (Bcl-2) alongside Bcl-2-associated × protein (Bax) overexpression [5,9].

Evidence exists that autophagy dysregulation is a crucial mechanism that mediates Cd-evoked testicular injury. Classically, autophagy is envisioned as a protective mechanism for cells to rid themselves of damaged mitochondria and misfolded proteins through the autophagosome–lysosome system [10,11,12]. When cells are under stress, autophagy serves as a pro-survival mechanism that prevents cell death and maintains cellular homeostasis [13,14]. Autophagy is limited under physiological settings; however, specific triggers, such as starvation, infection, and ischemia/hypoxia, have been identified as conditions that initiate autophagy. Autophagy flux comprises several steps which start with phagophore formation, an isolation membrane that surrounds the damaged targets. To engulf damaged organelles/proteins, the phagophore is then transformed into a double-membraned autophagosome [10,15]. Subsequently, autophagosome maturation takes place by the fusion of the autophagosome with the lysosome, generating an autophagolysosome, where lysosomal acid hydrolases degrade the engulfed material [15]. Generally, the pathology of testicular diseases reveals that hyperactive [14] as well as defective autophagic events [12,16] are described in the literature. In cadmium-induced testicular injury, impaired autophagy flux was detected even in the presence of increased expression of some autophagy signals [12,16,17]. In perspective, enhancement of lysosomal membrane permeability [18] and interference with calcium-dependent autophagosome–lysosome fusion [19] have been tightly linked to cadmium-evoked autophagy impairment. Notably, sequestome 1/protein 62 (SQSTM-1/p62) and Beclin 1 have been envisioned as reliable autophagy markers. In this regard, SQSTM-1/p62 aids in the clearance of ubiquitin-tagged proteins, and thus, its levels are increased in the case of defective autophagy. On the other hand, Beclin 1 plays a role in autophagosome production during the autophagic sequestration process. Hence, its levels are decreased in the case of impaired autophagy [12,16,17]. Of note, the autophagy events are positively driven by 5’ adenosine monophosphate-activated protein kinase (AMPK)/mammalian target of rapamycin (mTOR) pathway stimulation, which is brought about by increasing the p-AMPK/total AMPK ratio and lowering the p-mTOR/total mTOR ratio [16,20]. Interestingly, previous studies in the literature have described that cadmium-triggered testicular damage in rodents can be mitigated in part by activating AMPK/mTOR pathway [21]. 

In order to maintain tissue homeostasis, autophagy and apoptosis are tightly regulated [13,14]. While autophagy promotes the survival of cells when subjected to stresses, apoptosis serves as a death-favoring mechanism by killing severely damaged/mutated cells [10,13]. In various testicular diseases, the interaction between autophagy and apoptosis has been documented [12,21]. In this regard, autophagy has been shown to have a suppressive effect on apoptotic cell death in testicular dysfunction [12,22] alongside other experimental conditions, including neurological diseases [23] and hepatic injury [24].

**Figure 1:**
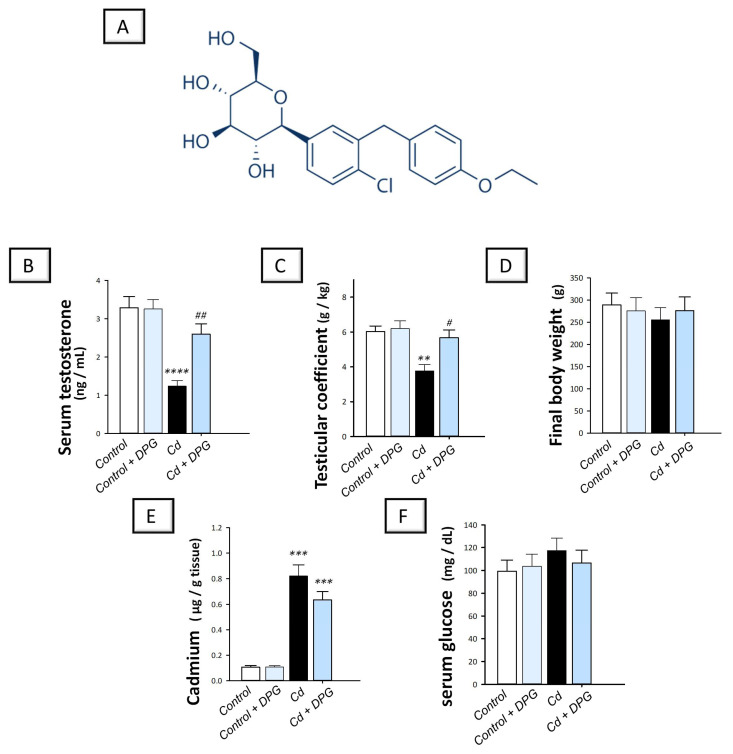
Dapagliflozin reduces the pathological symptoms of cadmium-evoked testicular damage in rats. The chemical structure of dapagliflozin is illustrated in (**A**). The structure was drawn using ChemDraw (PerkinElmer, Waltham, MA, USA). The beneficial effects of dapagliflozin in cadmium-intoxicated animals were revealed by its activity in augmenting serum testosterone levels (**B**) and increasing the testicular coefficient (**C**), with no significant effect on the final body weight of the animals (**D**), the Cd metal testicular content (**E**), or serum glucose levels (**F**). For *n* = 6, the results are displayed as the mean ± standard error of the mean. The testicular damage in rats was prompted by cadmium chloride administration (5 mg/kg/day) via the oral route for 60 days. Dapagliflozin (1 mg/kg/day) was given by oral gavage. At the end of the experiment, testicular tissues and sperms were harvested. Statistical significance: ** p < 0.01, *** p < 0.001, or **** p < 0.0001, compared with the vehicle-treated control group; *^#^ p* < 0.05, *^##^ p* < 0.01, compared with the cadmium chloride-treated group. DPG, dapagliflozin; Cd, cadmium chloride.

As a selective sodium–glucose co-transporter 2 (SGLT2) inhibitor, dapagliflozin (DPG; Forxiga^®^; the chemical structure is depicted in Figure 1A) has emerged as an effective therapy for type 2 diabetes with a diminished risk of hypoglycemia in the clinical setting [25]. From a mechanistic perspective, DPG controls type 2 diabetes mellitus by serving as a competitive and highly selective inhibitor of the SGLT2 transporter protein, which is expressed in renal tubular epithelial cells across the brush-border membrane of the proximal convoluted tubules. Hence, patients with type 2 diabetes benefit from DPG’s ability to increase glucose excretion in the urine and to improve both fasting and postprandial blood sugar levels [25,26]. In the preclinical setting, no safety concerns were reported for DPG in rats and beagle dogs, according to extensive non-clinical toxicology assessments in these species [27]. Notably, evidence exists that SGLT2 is mainly expressed by the kidney tissue with lower expression in the heart, salivary gland, liver, and thyroid. However, SGLT2 is minimally expressed by the testicular tissue, hence its physiological role is likely to be marginal [28,29,30]. Away from its SGLT2 inhibitory effects and glucose-lowering potential, DPG has demonstrated marked antioxidant [31,32,33,34], anti-apoptotic [31,33,34], and anti-inflammatory [31,32,34] features in the preclinical setting, as established in normoglycemic rodents. In fact, the antioxidant, anti-apoptotic, and anti-inflammatory features of DPG are the determining factors that mediate its efficacy for the attenuation of multiple pathological disorders, including experimental models of inflammatory bowel disease [31], coronary ligation-induced myocardial infarction [32], cardiac ischemia/reperfusion injury [34], gentamicin-induced nephrotoxicity [33], pentylenetetrazol (PTZ)-triggered epilepsy [35], rotenone-induced Parkinson’s disease [36], and chronic stress-triggered depression-like behavior [37]. Thus, the marked antioxidant, anti-apoptotic, and anti-inflammatory features of DPG encouraged us to examine its potential competence for mitigating cadmium-evoked testicular damage, a testicular pathology which is mediated by enhanced oxidative stress, apoptosis, and inflammation [1,14,38]. This may reveal additional clinical value for DPG in type 2 diabetes patients with cadmium-evoked testicular damage. Hence, the present study investigated the prospect of using DPG to counteract cadmium-induced testicular damage and spermatogenesis aberrations. The underlying molecular pathways of DPG were studied, particularly those related to oxidative stress, autophagy, and apoptosis, such as AMPK/mTOR cascade and SIRT1/Nrf2/HO-1 cytoprotective axis. Notably, in the present work, we used normoglycemic rats to examine DPG’s possible ameliorative impact on testicular tissue away from its glucose-lowering properties. This strategy is based on the notion that hyperglycemia has been linked to testicular dysfunction and sperm abnormalities [30]. In fact, the approach of using normoglycemic animals for investigating DPG actions has already been previously characterized in the literature, as described in experimental models of coronary ligation-induced myocardial infarction [32], cardiac ischemia/reperfusion injury [34], gentamicin-induced nephrotoxicity [33], PTZ-triggered epilepsy [35], rotenone-induced Parkinson’s disease [36], and chronic stress-triggered depression-like behavior [37].

## 2. Results

### 2.1. Dapagliflozin Improves Serum Testosterone and the Testicular Coefficient without Impacting Serum Glucose or Testicular Cadmium Uptake in Cd-Triggered Testicular Damage

The investigation of testicular dysfunction in animals was accomplished by determining the serum testosterone levels and the testicular coefficient (weight of the testes/total body weight of the animal). Meanwhile, the impact of DPG on testicular cadmium levels and serum glucose was examined. Compared to the control group, cadmium-intoxicated rats demonstrated a significant decrease in serum testosterone (*p* < 0.0001) by 62.4% and the testicular coefficient (*p* < 0.01) by 37.5%, and non-significant changes in final body weight of animals, as depicted in Figure 1. The co-treatment with DPG reversed these aberrations, including the elevation of serum testosterone levels (*p* < 0.01) by 110% and the testicular coefficient (*p* < 0.05) by 50.8% with no significant effect on the final body weight of animals. Concerning the testicular cadmium levels, cadmium-intoxicated rats demonstrated a significant increase in testicular Cd (*p* < 0.001) by 669.1%, compared to the control group; however, co-treatment with DPG prompted a non-significant change in testicular Cd. For serum glucose levels, no significant changes were detected among all the experimental groups. Together, these findings suggest that DPG can attenuate the disrupted testicular function without altering testicular Cd uptake or affecting serum glucose levels in cadmium-intoxicated rats.

### 2.2. Dapagliflozin Rescues Sperm Abnormalities and Enhances Sperm Characteristics in the Testicular Damage Triggered by Cd in Rats

**Figure 2:**
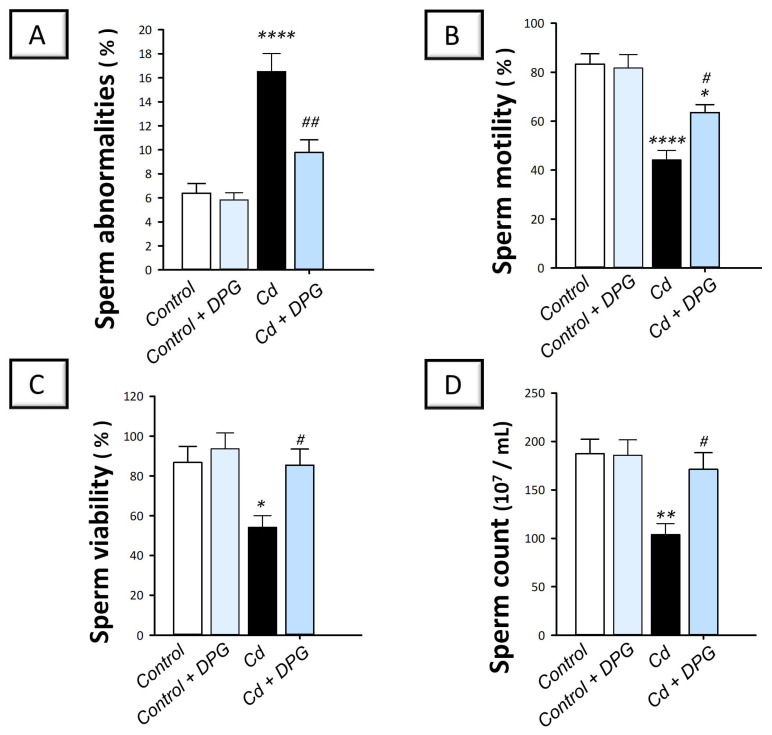
Dapagliflozin improves sperm characteristics in cadmium-evoked testicular damage in rats. The favorable outcomes of dapagliflozin were revealed by its activity in suppressing sperm abnormalities (**A**) as well as increasing sperm motility (**B**), sperm viability (**C**), and sperm count (**D**). For *n* = 6, the results are displayed as the mean ± standard error of the mean. The testicular damage in rats was prompted by cadmium chloride administration (5 mg/kg/day) via the oral route for 60 days. Dapagliflozin (1 mg/kg/day) was given by oral gavage. At the end of the experiment, testicular tissues and sperm were harvested. Statistical significance: * p < 0.05, ** p < 0.01, or **** p < 0.0001, compared with the vehicle-treated control group; *^#^ p* < 0.05, or *^##^ p* < 0.01, compared with the cadmium chloride-treated group. DPG, dapagliflozin; Cd, cadmium chloride.

The investigation of the testicular spermatogenesis process in animals was accomplished by determining several sperm parameters, including sperm abnormalities, motility, viability, and count. Compared to the control group, cadmium-intoxicated rats demonstrated a significant increase in sperm abnormalities (*p* < 0.0001) by 158.9% alongside a significant decline in motility (*p* < 0.0001) by 47%, viability (*p* < 0.05) by 37.6%, and sperm count (*p* < 0.01) by 44.5% (Figure 2). The co-treatment with DPG counteracted these perturbations, including the significant suppression of sperm abnormalities (*p* < 0.01) by 40.8% together with a significant increase in motility (*p* < 0.05) by 43.8%, viability (*p* < 0.05) by 57.7%, and sperm count (*p* < 0.05) by 64.6%. These findings suggest that DPG can mitigate disrupted spermatogenesis in cadmium-intoxicated rats.

### 2.3. Dapagliflozin Attenuates Testicular Histopathological Changes in Cd-Intoxicated Rats

**Figure 3:**
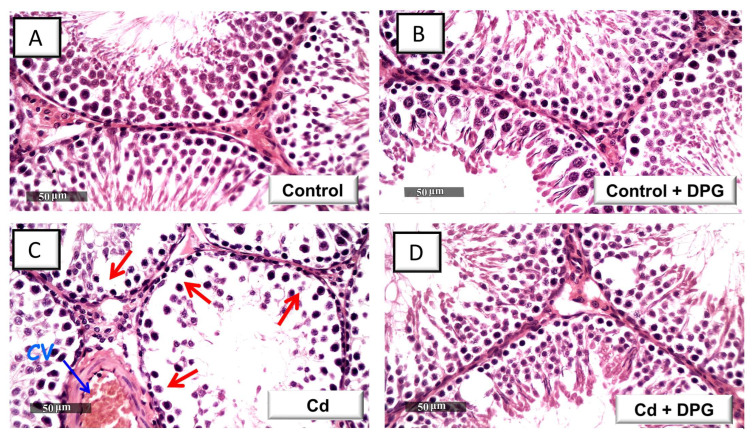

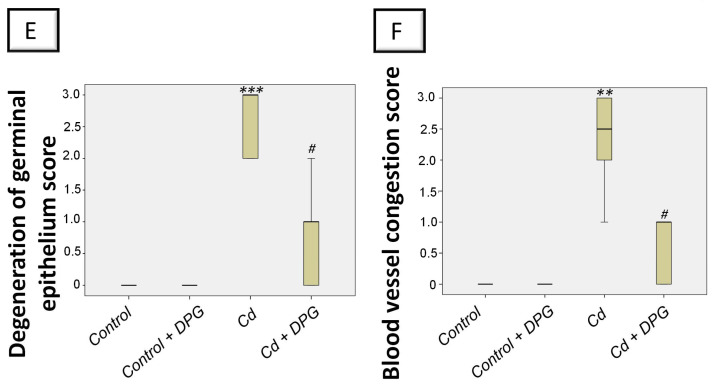
Dapagliflozin attenuates the testicular histopathological alterations in cadmium-evoked testicular damage in rats. Hematoxylin and eosin (H-E)-stained sections were used to study the histopathological changes in testicular specimens. Specimens of the control group (**A**) and the dapagliflozin-treated control group (**B**) demonstrated typical seminiferous tubules lined with intact germinal epithelium at several development stages and regular Sertoli cells. (**C**) The seminiferous tubules of cadmium-intoxicated rats show notable tubule disorganization, germinal cell degeneration (arrow), and marked blood vessel congestion (CV). (**D**) The histological abnormalities were reduced in the dapagliflozin-treated cadmium-intoxicated group, as seen by the normal structure of seminiferous tubules with typical germinal epithelium. (**E**,**F**) Scoring of germinal epithelium degeneration and blood vessel congestion, respectively. For *n* = 6, the results are displayed as the median with the interquartile range. ** p < 0.01, or *** p < 0.001, compared with the vehicle-treated control group; *^#^ p* < 0.05, compared with the cadmium chloride-treated group. DPG, dapagliflozin; Cd, cadmium chloride.

The damage of the testes was further investigated by microscopy to confirm the favorable effects of DPG against Cd-induced testicular damage. As demonstrated in Figure 3A,B, both the control and the DPG-treated control groups displayed normal testicular structure, including regular seminiferous tubules, well-organized germinal epithelium, and normal Sertoli cells. Compared to the control group, cadmium-intoxicated rats demonstrated a variety of histological abnormalities, such as spermatocyte degeneration and seminiferous tubule disorganization. There was also evidence of spermatid giant cell formation and congestion of interstitial blood vessels in the seminiferous tubules (Figure 3C). The co-treatment with DPG reduced these histological changes, as seen by the intact seminiferous tubules with typical epithelial cell maturation phases and ordered germinal epithelium (Figure 3D). Nonetheless, in the DPG-treated group, discrete regions of mild blood vessel occlusion and interstitial edema were still identified. These findings were reinforced by the scoring of the histological lesions (Figure 3E,F) where significant increases in the scores of testicular germinal epithelium degeneration and blood vessel congestion were revealed in the cadmium-intoxicated group compared to the control group. Favorably, the damage scores were attenuated by DPG administration, confirming DPG’s ability to mitigate the testicular damage.

### 2.4. Dapagliflozin Activates SIRT1/Nrf2/HO-1 Axis and Lowers Testicular Oxidative Insult in the Testicular Damage Triggered by Cd in Rats

**Figure 4:**
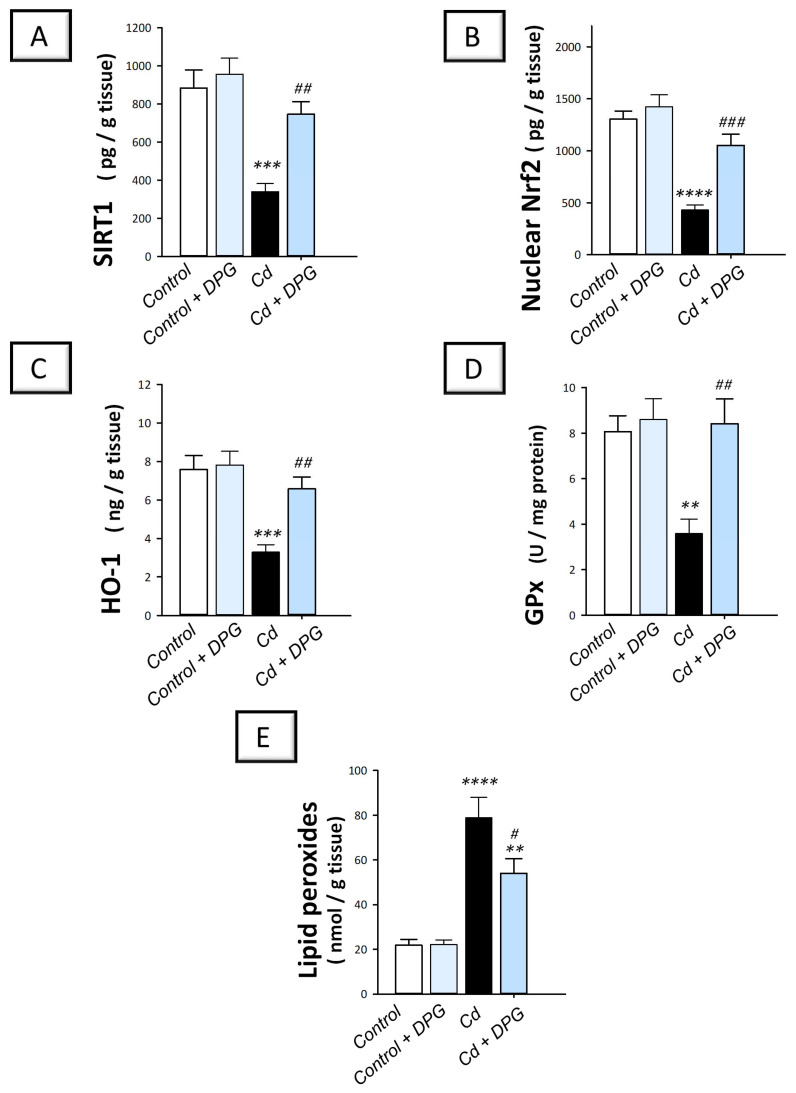
Dapagliflozin activates the testicular SIRT1/Nrf2/HO-1 cascade and lowers lipid peroxides in the testicular damage triggered by Cd in rats. The favorable outcomes of dapagliflozin were revealed by activation of the testicular SIRT1/Nrf2/HO-1 cascade, as seen by increases in SIRT1 (**A**), Nrf2 nuclear levels (**B**), HO-1 (**C**), and GPx (**D**). Meanwhile, it lowered lipid peroxides levels (**E**). For *n* = 6, the results are displayed as the mean ± standard error of the mean. ** p < 0.01, *** p < 0.001, or **** p < 0.0001, compared with the vehicle-treated control group; *^#^ p* < 0.05, *^##^ p* < 0.01, or *^###^ p* < 0.001, compared with the cadmium chloride-treated group. DPG, dapagliflozin; Cd, cadmium chloride.

The impact of DPG on the testicular redox changes instigated by cadmium was studied by determining the lipid peroxide levels and changes in the antioxidant SIRT1/Nrf2/HO-1 pathway. Compared to the control group, the testicular tissues of cadmium-intoxicated rats demonstrated exaggerated oxidative perturbations manifested by a significant decline in SIRT1 (*p* < 0.001), nuclear Nrf2 nuclear (*p* < 0.0001), HO-1 (*p* < 0.001), and GPx (*p* < 0.01) by 61.6%, 67%, 56.7%, and 55.5%, respectively, alongside a significant elevation in lipid peroxides (*p* < 0.0011) by 261.6% (Figure 4A–E). The co-treatment with DPG of cadmium-intoxicated rats significantly augmented the protein expression of SIRT1 (*p* < 0.01), Nrf2 (*p* < 0.001), HO-1 (*p* < 0.01), and GPx (*p* < 0.01) by 119.6%, 144.4%, 100.1%, and 134.3%, respectively, and significantly diminished the testicular lipid peroxide levels (*p* < 0.05) by 31.5%. These findings suggest that DPG can stimulate SIRT1/Nrf2/HO-1 axis in cadmium-intoxicated rats.

### 2.5. Dapagliflozin Inhibits Pro-Apoptotic Events in the Testicular Damage Triggered by Cd in Rats

**Figure 5:**
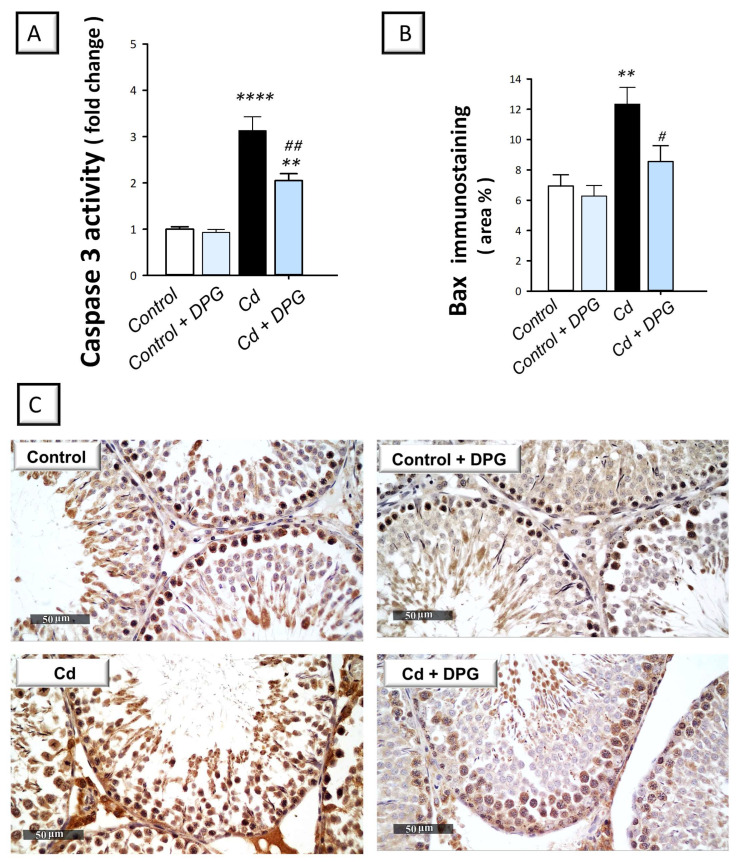
Dapagliflozin dampens the apoptotic events by lowering caspase-3 activity and Bax protein expression in the testicular damage triggered by Cd in rats. (**A**) The activity of caspase-3. (**B**) The graph illustrates the quantitative analysis of Bax immunoreactivity expressed as the area percentage. In each group, six non-overlapping fields were examined. (**C**) Representative images for Bax immunoreactivity (photomicrographs at 400× magnification). In the control and dapagliflozin-treated control groups, respectively, the immunoreactivity (brown color) of testicular Bax was minimal. In the cadmium-intoxicated group, Bax immunoreactivity was increased. In cadmium-intoxicated rats, dapagliflozin attenuated testicular Bax immunoreactivity. For *n* = 6, the results are displayed as the mean ± standard error of the mean. ** p < 0.01, or **** p < 0.0001, compared with the vehicle-treated control group; *^#^ p* < 0.05, *^##^ p* < 0.01, compared with the cadmium chloride-treated group. DPG, dapagliflozin; Cd, cadmium chloride.

**Figure 6:**
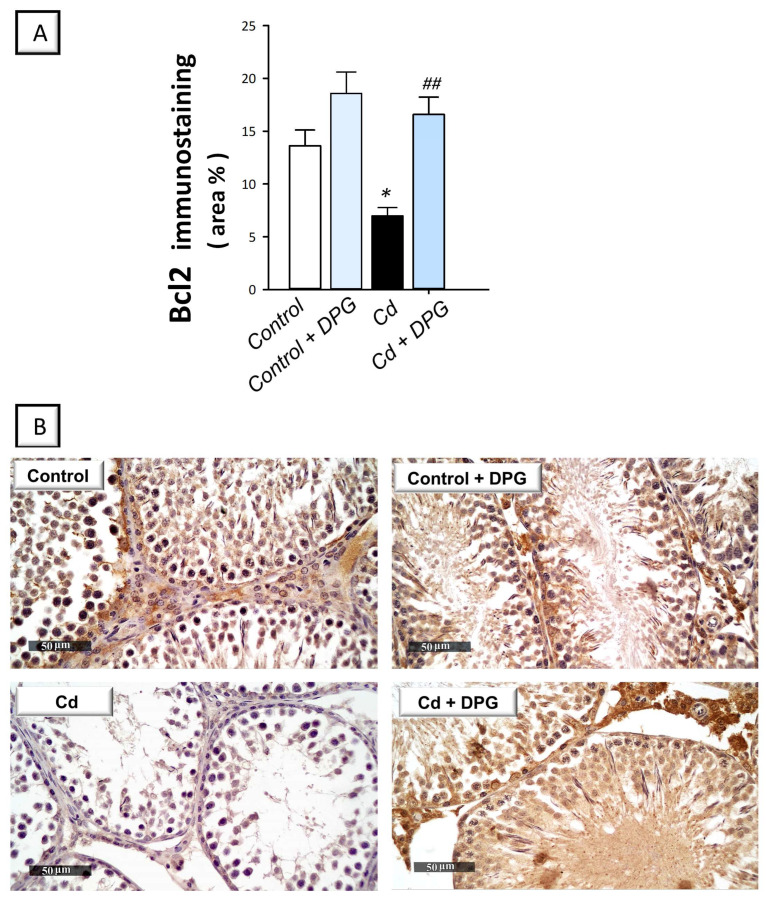
Dapagliflozin augments Bcl-2 protein expression in the testicular damage triggered by Cd in rats. (**A**) The graph illustrates the quantitative analysis of Bcl2 immunoreactivity expressed as the area percentage. In each group, six non-overlapping fields were examined. (**B**) Representative images for Bcl2 immunoreactivity (photomicrographs at 400× magnification). In the control and dapagliflozin-treated control groups, respectively, the immunoreactivity (brown color) of testicular Bcl-2 was intense. In the cadmium-intoxicated group, Bcl-2 immunoreactivity was suppressed. In cadmium-intoxicated rats, dapagliflozin administration increased the testicular immunoreactivity of Bcl-2. For *n* = 6, the results are displayed as the mean ± standard error of the mean. * p < 0.05, compared with the vehicle-treated control group; *^##^ p* < 0.01, compared with the cadmium chloride-treated group. DPG, dapagliflozin; Cd, cadmium chloride.

In testicular pathologies, inhibition of autophagy flux has been associated with the stimulation of the pro-apoptotic machinery [21,38]. Hence, testicular apoptosis was studied by quantifying the activity of caspase-3 and the protein expression of Bcl-2 and Bax by immunohistochemistry. Compared to the control group, the testicular tissues of cadmium-intoxicated rats demonstrated marked pro-apoptotic events evidenced by a significant increase in caspase-3 activity (*p* < 0.0001) by 212.8% and Bax immunoreactivity (*p* < 0.01) by 78% (Figure 5A,B). In tandem, the testicular tissues of cadmium-intoxicated rats demonstrated a significant decrease in Bcl-2 immunoreactivity (*p* < 0.05) by 48.7% (Figure 6A). The co-treatment with DPG of cadmium-intoxicated rats counteracted the activation of the apoptotic machinery, as demonstrated by a significant decrease in caspase-3 activity (*p* < 0.01) by 34.6% and Bax immunoreactivity (*p* < 0.05) by 30.8%, alongside a significant increase in Bcl-2 immunoreactivity (*p* < 0.01) by 137.6%. Together, these findings show that the ability of DPG to suppress testicular apoptosis contributes, at least in part, to the attenuation of testicular damage in cadmium-intoxicated rats. 

### 2.6. Dapagliflozin Rescues Defective Testicular Autophagy by Lowering the Accumulation of SQSTM-1/p62 and Upregulating Beclin 1 in the Testicular Damage Triggered by Cd in Rats

**Figure 7:**
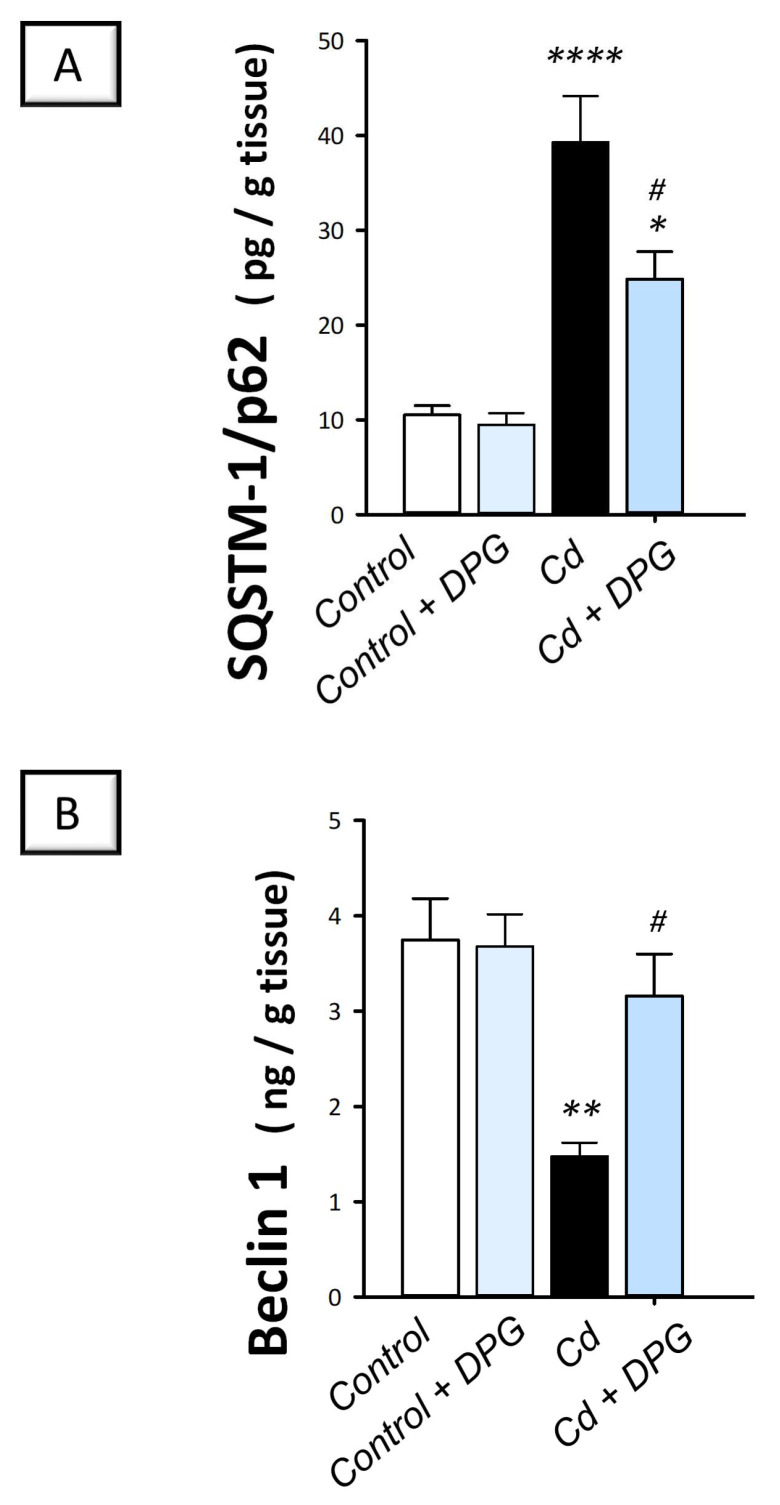
Dapagliflozin downregulates the protein expression of SQSTM-1/p62 and upregulates Beclin 1 in the testicular damage triggered by Cd in rats. Dapagliflozin enhanced testicular autophagy by lowering SQSTM-1/p62 protein accumulation (**A**) and boosting Beclin1 protein expression (**B**). For *n* = 6, the results are displayed as the mean ± standard error of the mean. * p < 0.05, ** p < 0.01, or **** p < 0.0001, compared with the vehicle-treated control group; *^#^ p* < 0.05, compared with the cadmium chloride-treated group. DPG, dapagliflozin; Cd, cadmium chloride.

Herein, we investigated whether the promising ameliorative effects of DPG were linked to autophagy. Hence, the protein expression of SQSTM-1/p62 was studied as a marker of defective autophagy that characterizes impaired autophagosome breakdown. Meanwhile, Beclin 1 was examined as a reliable positive marker of autophagy [12,16]. Compared to the control group, the testicular tissues of cadmium-intoxicated rats demonstrated defective autophagy that was marked by a significant increase in SQSTM-1/p62 protein levels (*p* < 0.0001) by 274.1% together with a significant decrease in Beclin 1 protein expression (*p* < 0.01) by 60.6% (Figure 7). The co-treatment with DPG of cadmium-intoxicated rats rescued the impairment in autophagy, which was manifested by a significant decrease in SQSTM-1/p62 protein levels (*p* < 0.05) by 36.7% alongside a significant increase in Beclin 1 protein expression (*p* < 0.05) by 113.9%. Together, these findings show that the ability of DPG to stimulate testicular autophagy contributes, at least in part, to the attenuation of testicular damage in cadmium-intoxicated rats. 

### 2.7. Dapagliflozin Stimulates the Autophagy-Associated AMPK/mTOR Pathway in the Testicular Damage Triggered by Cd in Rats

**Figure 8:**
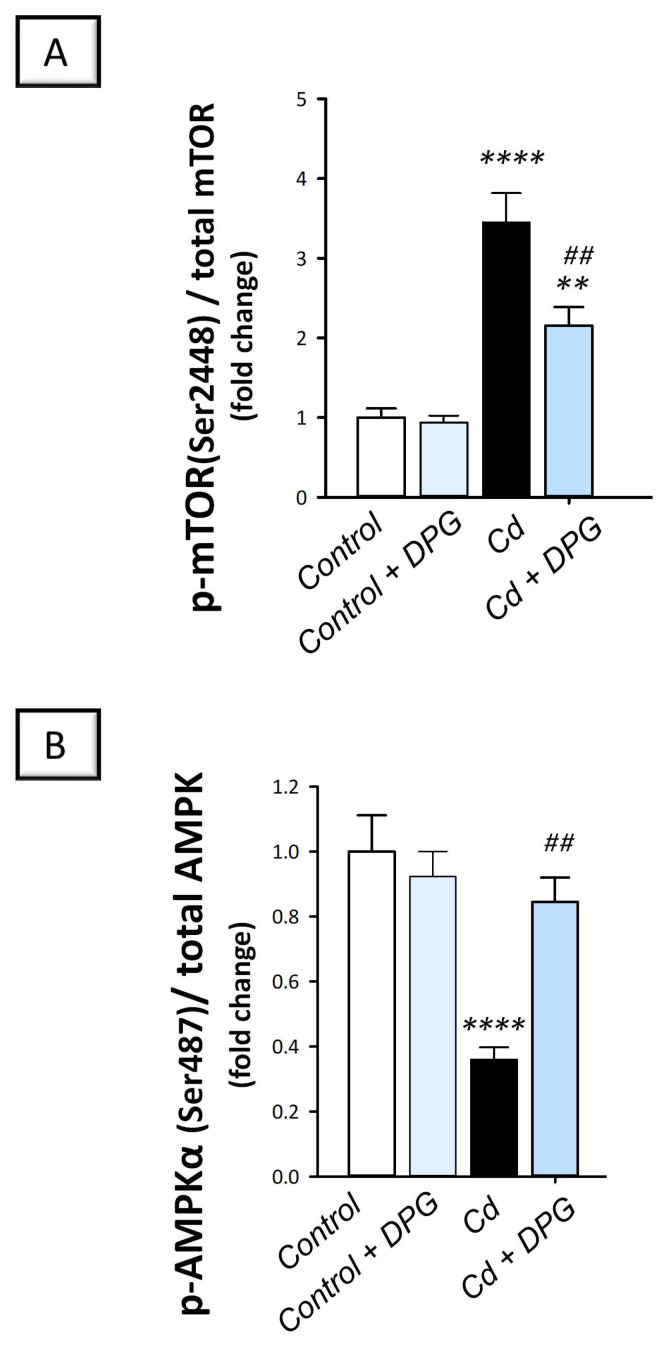
Dapagliflozin activates the testicular AMPK/mTOR pathway in the testicular damage triggered by Cd in rats. Dapagliflozin stimulated the autophagy-associated AMPK/mTOR pathway as seen by a decrease in the p-mTOR (Ser2448)/total mTOR ratio (**A**) together with an increase in the p-AMPK (Ser487)/total AMPK ratio (**B**). For *n* = 6, the results are displayed as the mean ± standard error of the mean. ** p < 0.01, or **** p < 0.0001, compared with the vehicle-treated control group; *^##^ p* < 0.01, compared with the cadmium chloride-treated group. DPG, dapagliflozin; Cd, cadmium chloride.

The testicular pro-autophagy AMPK/mTOR pathway was explored to further define the autophagy events [14,16]. To this end, the detection of p-AMPK (Ser487)/total AMPK and p-mTOR (Ser2448)/total mTOR signals was applied. Compared to the control group, the testicular AMPK/mTOR pathway was inhibited in cadmium-intoxicated rats. This was marked by a significant increase in the p-mTOR (Ser2448)/total mTOR ratio (*p* < 0.0001), a negative autophagy signal, by 244.9% together with a significant decline in p-AMPK (Ser487)/total AMPK ratio (*p* < 0.0001) by 64% (Figure 8). The co-treatment with DPG of cadmium-intoxicated rats counteracted these changes and stimulated AMPK/mTOR pathway, which was manifested by a significant decrease in the p-mTOR (Ser2448)/total mTOR ratio (*p* < 0.01) by 37.5% alongside a significant increase in the p-AMPK (Ser487)/total AMPK ratio (*p* < 0.01) by 135%. Together, these findings show that the ability of DPG to stimulate testicular AMPK/mTOR contributes, at least in part, to the attenuation of testicular damage in cadmium-intoxicated rats.

## 3. Discussion

**Figure 9:**
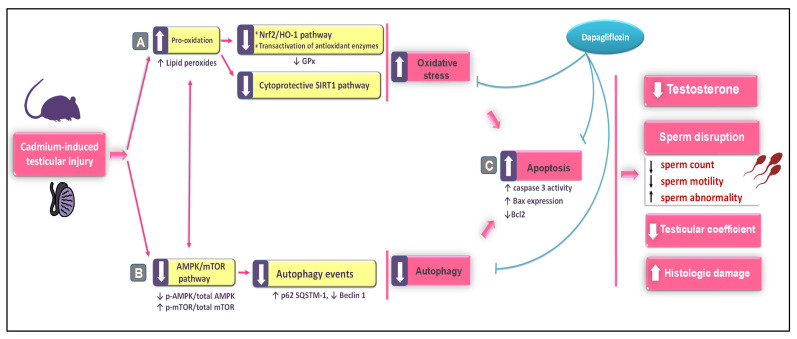
A concise outline for the molecular events underpinning dapagliflozin’s mitigating impact against the testicular damage triggered by Cd in rats. The testicular impairment and spermatogenesis derangement were improved with dapagliflozin by (**A**) Stimulation of the testicular SIRT1/Nrf2/HO-1 cytoprotective cascade, augmentation of antioxidants, and curtailment of oxidative events. (**B**) Activation of AMPK/mTOR pathway, as evidenced by the decrease in the p-mTOR/total mTOR ratio and the concurrent increase in the p-AMPK/total AMPK ratio. These events were associated with an enhancement of autophagy that was manifested by a lowering in SQSTM-1/p62 accumulation and an increase in Beclin 1. (**C**) Suppression of testicular apoptotic machinery, as evidenced by caspase-3 activity inhibition, Bax downregulation, and Bcl2 upregulation. Activation is illustrated by a solid arrow, while inhibition is illustrated by a blunt arrow.

The present study proves that DPG can protect against cadmium-induced testicular damage by activating testicular autophagy while suppressing apoptotic cell death and oxidative events. At the molecular level, stimulation of the pro-autophagy AMPK/mTOR cascade and augmentation of the cytoprotective SIRT1/Nrf2/HO-1 axis were implicated in mediating DPG’s promising effects (Figure 9).

Autophagy is crucial for cellular homeostasis as a catabolic pathway that eliminates misfolded proteins and damaged organelles from stressed cells. These events take place via the autophagy–lysosome pathway where the damaged organelles/proteins reach the lysosome for destruction [10,11,13]. When cells are subjected to stresses, autophagy aims at advocating cellular survival [14]. In perspective, the removal of damaged mitochondria, the primary ROS generation source in cells, is a cardinal event for dampening oxidative stress and maintaining cellular homeostasis [9,12]. In the context of testicular pathologies, the literature has described defective [12,16,21] and overactive autophagy [14]. Likewise, in cadmium-triggered testicular injury models, autophagy defects [12,21] as well as overactivity [39] have been reported. Hence, further exploration of the role of autophagy in cadmium-induced testicular pathogenesis was warranted. Herein, the current findings have shown that cadmium results in defective testicular autophagy, as revealed by accumulated SQSTM-1/p62 together with downregulated Beclin 1 in rat testicular tissues. Indeed, the pathogenesis of cadmium-evoked testicular damage revealed that autophagy dysfunction is tightly linked to testicular damage and decreased spermatogenesis [12,21]. Autophagy impairment is driven by cadmium’s ability to interfere with the calcium-associated fusion of lysosomes with autophagosomes [19] and to increase lysosomal membrane permeability/damage [18]. In fact, defective autophagy results in cellular accumulation of ROS/damaged mitochondria, instigating the signaling of apoptotic cell death [16]. The latter event is marked by caspase-3 activation, which has been reported to block autophagy initiation through caspase-3-mediated Beclin 1 degradation [40]. 

Evidence exists that the interventions that enhance germ cell autophagy can attenuate cadmium-evoked testicular dysfunction [12,21,41]. In rodent models of testicular damage, autophagy stimulation has been shown to dampen pro-apoptotic events [12,16,41]. As a pro-survival mechanism against cellular stressors, the autophagy process maintains cellular energy production through the autophagy-mediated supply of recycled metabolic substrates [10,11]. In accordance with these data, the current experiments have revealed the ability of DPG to activate the testicular autophagy machinery that was marked by the diminished accumulation of SQSTM-1/p62 and Beclin 1 upregulation. In the context of autophagy, Beclin 1 is involved in the production of autophagosomes during the autophagic sequestration stage, which stimulates autophagy [14]. Similarly, SQSTM-1/p62 detects ubiquitin-tagged proteins to aid their clearance, and itself is degraded during autophagy. Hence, it serves as a negative autophagy marker [10,14]. In fact, the stimulation of autophagy has been previously characterized for DPG as an underlying mechanism for mitigating trinitrobenzene-induced colonic injury [31], renal injury in high-fat diet-induced obese rats [42], and hepatic steatosis [43]. The observed autophagy stimulation is likely implicated in serum testosterone enhancement by DPG. The association between testosterone synthesis and testicular autophagy has been elucidated, since autophagy has previously been reported to promote testosterone synthesis in Leydig cells via an increase in the uptake of cholesterol into Leydig cells, favoring steroidogenesis [44]. In perspective, the interaction between the lysosome system and autophagosome-interceded cholesterol trafficking supports the effective delivery of cholesterol for steroidogenesis [44,45]. Moreover, the observed decline in testicular oxidative stress by DPG, likely driven by autophagy-linked elimination of damaged mitochondria/ROS, has the potential to restore steroidogenic enzymes, thereby augmenting testosterone production [16]. 

The current findings have also revealed inhibition of the testicular AMPK/mTOR pathway, a positive regulator of autophagy, in response to cadmium. This was manifested by an upregulated expression of p-mTOR together with a downregulated expression of p-AMPK. Consistently, previous studies have revealed suppression of AMPK/mTOR in lipopolysaccharide-evoked Leydig cell injury in vitro [16] and testicular damage in experimental models [14]. Herein, DPG stimulated the testicular AMPK/mTOR pathway, as marked by the increased p-AMPK/total AMPK and decreased p-mTOR/total mTOR levels driving autophagy activation. Indeed, AMPK/mTOR pathway activation has been demonstrated to mediate favorable autophagy stimulation and associated mitigation of cadmium-triggered testicular damage in rodents [21] and LPS-induced Leydig cell injury [16]. Evidence exists that the low energy sensor AMPK can maintain cellular energy generation by stimulating autophagy events through dampening of p-mTOR/total mTOR ratio [16]. Interestingly, AMPK has been reported to control spermatozoa quality and functions [46]. Through the regulation of spermatozoa motility, acrosome reaction, and the proliferation of somatic cells (Sertoli and Leydig) in the testis, AMPK has been reported to control the quality of spermatozoa. In addition, AMPK dampens lipid peroxidation and boosts several antioxidant enzymes to improve spermatozoa quality [16,46]. In the context of DPG’s favorable outcomes in the previous literature, AMPK/mTOR pathway activation has been previously reported as an underlying mechanism for DPG to attenuate experimental inflammatory bowel disease [31]. 

Repeated exposure to cadmium has been shown to drive testicular apoptosis mainly via the mitochondrial pathway [1,14,38]. In fact, mitochondrial membrane integrity is maintained by the balance between the pro-apoptotic Bax and the anti-apoptotic Bcl-2 [14]. In line with these reports, the current findings show that cadmium stimulated testicular pro-apoptotic events, as seen by Bax overexpression and Bcl-2 downregulation, an event that culminates in caspase-3 activation [1,14]. The observed cadmium-induced autophagy blockade, marked by SQSTM-1/p62 accumulation, has been shown to switch on the pro-apoptotic events [21,24]. In fact, the observed testicular oxidative stress can trigger testicular apoptosis. This is consistent with the growing body of evidence that oxidative stress and exaggerated ROS production have been associated with apoptotic cell death and the linked derangement of spermatogenesis and testicular junction proteins [47]. Herein, DPG counteracted the pro-apoptotic events as seen by Bcl-2 upregulation and Bax downregulation. These favorable events are associated with autophagy reinforcement and spermatogonial cell proliferation [48,49]. Notably, autophagy activation in Sertoli cells has been reported to inhibit cadmium-evoked germ cell apoptosis [38]. 

Oxidative stress, principally targeting germ cells/spermatozoa and Leydig cells, is tightly associated with the pathology of cadmium-evoked testicular damage [1,5,7,12,22], which concurs with the present study findings. At the molecular level, cadmium triggers oxidative derangements by (A) binding to the thiol moiety and inactivation of cellular antioxidants, such as reduced glutathione; (B) disturbance of the mitochondrial respiratory chain and associated ROS overshooting; (C) inactivation of antioxidant enzymes, such as GPx, by interference with their cofactor metal ions [5,7,12]; and (D) deregulation of testicular extracellular matrix turnover due to interference with the essential metal ions required for matrix metalloproteinases [50]. The present work further demonstrated that cadmium triggered an inactivation of SIRT1/Nrf2/HO-1 cascade in rat testicular tissue, events which were reversed by DPG. In perspective, SIRT1 is thought to play a critical role in spermatogenesis by modulating male germ cells, Leydig cells, and Sertoli cells, as proven in transgenic mice models [51]. The favorable effects of SIRT1 are due to oxidative insult suppression, chromatin remodeling, energy homeostasis, and mitochondrial biogenesis [7,51]. As regards oxidative events, SIRT1 has been reported to deacetylate/activate Nrf2, a critical antioxidant signal that controls the transcription of several antioxidant signals, including HO-1 and GPx. Indeed, the literature reveals that the therapeutic agents that can upregulate Nrf2/HO-1 axis have been previously demonstrated to attenuate cadmium-evoked testicular damage [7,8]. Meanwhile, SIRT1 has been demonstrated to suppress mTOR phosphorylation, driving AMPK/mTOR-associated autophagy stimulation and removal of damaged mitochondria [52]. Consistently, the antioxidant features of DPG have been previously shown to mediate DPG’s efficacy in combating experimental inflammatory bowel disease [31], cardiac ischemia/reperfusion injury [34], gentamicin-induced nephrotoxicity [33], and rotenone-induced PD [31]. Evidence has also revealed the crosstalk between autophagy flux and Nrf2/HO-1 pathway where Nrf2 serves as a transcription factor for the overexpression of SQSTM-1/p62 [53]. Likewise, Nrf2 has been implicated in the transcription of Atg3, Atg5, Atg7, and LAMP2A, which are essential genes for autophagy [54]. 

## 4. Materials and Methods

### 4.1. Chemicals

Sigma-Aldrich (St. Louis, MO, USA) provided the cadmium chloride (CdCl_2_), and AstraZeneca Pharmaceutical Company (Cairo, Egypt) provided dapagliflozin. The current study’s assays were carried out with the highest quality materials.

### 4.2. Animals

In the present work, male Sprague–Dawley rats (170–210 g) were procured from the Egyptian Drug Authority (EDA) animal breeding unit (Giza, Egypt). Controlled conditions were applied for animals, including constant temperatures (22 ± 2 °C), 12 h day/12 h night cycle, and 55% relative humidity. During the study period, unrestricted access to animal laboratory chow pellets and drinking water was permitted. Moreover, a span of 2-week acclimatization was executed before starting the experimental procedures. All protocols involving animal handling were carried out in accordance with the EDA’s standard protocols and the Laboratory Animals Care and Use Guide (NIH, Publication No. 85-23). Approval of the study protocol was obtained from the EDA’s Research Ethics Committee for Experimental and Clinical Studies under the permit number (Approval # NODCAR/I/33/2022). 

### 4.3. Experimental Design

**Figure 10:**
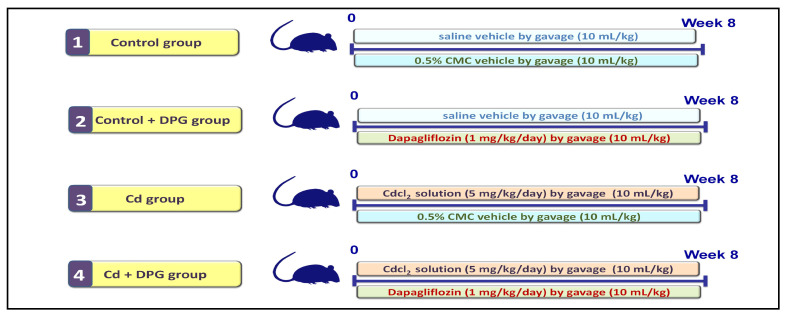
Summary of the experimental design.

In the present investigation, 24 rats were randomly allocated into four groups (*n* = 6) by a technician unaware of the study design, as summarized in Figure 10. In group I (Control group; *n* = 6), the rats received the vehicle for cadmium chloride by gavage (normal saline; 10 mL/kg/day). The animals in this group also received the oral vehicle of dapagliflozin (0.5% carboxymethyl cellulose; CMC) by gavage. These doses lasted for 8 weeks. In group II (Control + DPG group; *n* = 6), the rats received normal saline (10 mL/kg/day). The animals in this group also received dapagliflozin (1 mg/kg/day suspended in 0.5% CMC) by gavage. These doses lasted for 8 weeks. In group III (Cd group; *n* = 6), the rats received cadmium chloride solution (5 mg/kg/day) by gavage (dissolved in normal saline; 10 mL/kg/day). The animals in this group also received 0.5% CMC by gavage. These doses lasted for 8 weeks. The current experimental protocol is consistent with past literature [21,55]. In group IV (Cd + DPG group; *n* = 6), the rats received cadmium chloride solution (5 mg/kg/day) by gavage (10 mL/kg/day). The animals in this group also received dapagliflozin (1 mg/kg/day) by gavage. These doses lasted for 8 weeks. Herein, the dose of dapagliflozin was received 1 h after cadmium chloride administration.

The dose of dapagliflozin was chosen based on earlier research that showed its efficacy for mitigation of experimental models of inflammatory bowel disease [31], coronary ligation-induced myocardial infarction [32], cardiac ischemia/reperfusion injury [34], gentamicin-induced nephrotoxicity [33], PTZ-triggered epilepsy [35], rotenone-induced Parkinson’s disease [36], and chronic stress-triggered depression-like behavior [37]. The dose of dapagliflozin was also chosen according to the human equivalent dose (HED) calculation approach [56] in accordance with its frequently used dose in clinical practice. 

At the end of the experiment, rats were fasted overnight, and their body weight was recorded. After anesthesia with thiopental sodium solution intraperitoneally (30 mg/kg), blood was withdrawn from the retro-orbital plexus, and serum was isolated. Then, animal sacrifice was performed by cervical dislocation under anesthesia. Dissection of male reproductive organs was executed and the weight of the 2 testes was recorded for each animal. The cauda epididymis was cut, and the semen fluid was quickly extracted for examination. For histopathology examination and immunohistochemical detection, the left testis was preserved in 10% formol saline (3 specimens per group, randomly chosen). After decapsulation, the right testis was stored at −80 °C. For biochemical assays, one part of the right testis underwent lysis in protease/phosphatase-supplemented lysis solution (Tris, pH 7.4 (10 mM), glycerol (10%), EDTA (5 mM), and NaCl (200 mM)) for the ELISA studies. After centrifugation (10,000× *g* for 15 min at 4 °C), the supernatant was stored at −80 °C for further processing. The testosterone level was determined in the serum.

### 4.4. Examination of Sperm Characteristics

The examination of seminal fluid was executed as described [57]. Firstly, calculation of the sperm count was applied by chopping the epididymis to collect the seminal fluid into saline at 37 °C. A hemocytometer was used to count the sperms under a light microscope (at 400× magnification). Secondly, sperm motility was determined by adding one drop of 37 °C-preheated 2.9% sodium citrate to the seminal fluid. Within 2–4 min, the sperm suspension (50 µL) was added on a glass slide and the sperm motility was determined in 10 microscopical fields. Herein, the percentage of motile sperm relative to the total sperm number was determined. Finally, for sperm viability and morphological anomalies, one drop of the epididymal content was combined with one drop of Eosin–Negrosin stain. Three hundred sperms were inspected randomly at 400× magnification and the percentage of vital sperms (where the sperm head is not stained with Eosin–Negrosin stain) was recorded. Regarding sperm morphological anomalies, the total percentage of sperm abnormalities was recorded as previously described [58]. In this regard, the sperm abnormalities included anomalies in the sperm head as well as in the tail. The head anomalies comprised amorphous head, banana-shaped, hookless, and double-headed sperm whereas the tail anomalies comprised folded, bent, coiled, and double-tailed sperm. 

### 4.5. Determination of the Testicular Coefficient

As previously described [59], the testicular coefficient was computed as the two testes’ weight in grams divided by the final body weight in kg. 

### 4.6. Determination of Serum Testosterone

The levels of serum testosterone were measured with the aid of a Cusabio ELISA kit as instructed by the vendor (Catalog no. CSB-E05100r, Cusabio Technology, Houston, TX, USA). The final absorbance measurement of testosterone was applied at 450 nm.

### 4.7. Determination of Serum Glucose and Testicular Cadmium

Under the instructions of the provider, HUMAN Diagnostics (Wiesbaden, Germany, Cat. no. BD184), serum glucose was measured using a colorimetric kit. The final color’s O.D. was measured at 500 nm. As previously described [60], cadmium testicular content was measured. To this end, part of the testis was digested in 1 M nitric acid. After that, tissue ashing was carried out at 150 °C. A graphite furnace and PerkinElmer 3100 Atomic Absorption Spectrophotometer (Artisan Technology Group, Champaign, IL, USA) were used to detect the cadmium concentration at 228.8 nm. 

### 4.8. Determination of Testicular Oxidative Stress and Apoptosis

With the aid of a thiobarbituric acid assay, the content of malondialdehyde (MDA), an end product of lipid peroxides, was assessed in the testes, as described in [61], and the final absorbance was recorded at 535 nm. Rat-specific ELISA kits for SIRT1 (AFG Bioscience, Northbrook, IL, USA, Cat. no. EK720561), Nrf2 (Cat. no. MBS012148), and HO-1 (Elabscience, Wuhan, China, Cat. no. E-EL-R0488) [62] were used for the assay of each corresponding target. The Nrf2 content was examined in the testicular nuclear extract that was prepared with the aid of a Cayman nuclear extraction kit (Cayman Chemical, Ann Arbor, MA, USA, Cat. No. 10009277). The final absorbance measurement for SIRT1, Nrf2, and HO-1 was applied at 450 nm. The activity of GPx was attained using a Sigma-Aldrich colorimetric kit (Sigma-Aldrich, St. Louis, MO, USA, Cat. no. CGP1). To this end, monitoring the absorbance decline was applied at 340 nm using a kinetic program. Testicular caspase-3 activity was measured with the aid of the corresponding Sigma-Aldrich colorimetric assay kit according to the manufacturer’s instructions (Cat. no. CASP-3-C; Sigma-Aldrich, USA). At 405 nanometers, the O.D. of the final color was read.

### 4.9. Evaluation of Autophagy Markers

Commercial ELISA kits from MyBioSource were used to quantify the levels of Beclin 1 (Cusabio Technology, Houston, TX, USA, Cat. no. EL002658RA) and SQSTM-1/p62 (MyBioSource, San Diego, CA, USA, Cat. no. MBS3809397) as reliable autophagy markers, according to the manufacturer’s recommendations. For these autophagy markers, the final color was measured at 450 nm. The testicular expression of p-mTOR (Ser2448; Cat. no. 7976C) and total mTOR (Cell Signaling Technology, Danvers, MA, USA, Cat. no. 7974C) was carried out with the aid of the corresponding ELISA kits, as guided by the vendor’s instructions. The levels of p-AMPK(Ser487)/total AMPK were also evaluated using an ELISA kit from RayBiotech (Norcross, Peachtree Corners, GA, USA, Cat. no. PEL-AMPKA-S487-T). At 450 nm, the final color was measured.

### 4.10. Histopathology

Following testis dissection, fixation of the left testis in 10% formalin solution was performed for 24 h. A routine histology protocol was followed for washing, dehydration, and paraffin embedding [63,64]. Hematoxylin and eosin staining of 5-micron sections was performed. The tissue sections were inspected under a light microscope (Leica Microsystems GmbH, Wetzlar, Germany) and the digital pictures were obtained using a fitted digital camera. Scoring for the severity of the detected histopathological damage in the testicular tissue was applied, including the germinal epithelium degeneration and blood vessel congestion (*n* = 6 non-overlapping fields from 3 specimens per group). The scoring was applied according to a 0–4 system [65]; a score of 4 indicates degeneration affecting more than 60% of the examined testicular sections; a score of 3 indicates degeneration affecting 40–59% of the examined testicular sections; a score of 2 indicates degeneration affecting 20–39% of the examined testicular sections, a score of 1 indicates degeneration affecting less than 20% of the examined testicular sections; a score of 0 indicates no obvious pathological findings in the testicular sections.

### 4.11. Immunohistochemistry

As previously characterized, an immunohistochemistry protocol was implemented [66,67]. The 5-micron testicular cross-sections were treated with H_2_O_2_ for the inactivation of endogenous peroxidase activity. Antigen retrieval was applied with citrate buffer (pH 6.0) in the microwave, and section blockade with 5% bovine serum albumin was performed. The 5-micron sections were incubated at 4 °C overnight in humidified chambers with the specific primary antibodies: anti-Bcl-2 (Thermo Fisher Scientific, Waltham, MA, USA, 1:100 dilution; Cat. no. PA1-30411), or anti-Bax (Thermo Fisher Scientific, Waltham, MA, USA, 1:100 dilution, Cat. no. MA5-14003). The specific binding of primary antibodies was ensured by running negative controls where normal rabbit serum was applied instead of the corresponding antibody. The HRP-tagged secondary antibody was then applied to tissue sections for 30 min (DAKO’s HRP Envision kit, DAKO, Carpinteria, CA, USA), and the brown color was obtained using a DAB peroxidase substrate kit for 10 min. Hematoxylin was used to counterstain the sections and a Leica light microscope was used to examine/capture images (Leica Microsystems GmbH, Germany). The software of the Leica Application Module (Leica Microsystems GmbH, Germany) was used to quantify the immunoreactivity % for Bax and Bcl2 target proteins. Bias was avoided by making the specimen labeling anonymous for the observer. 

### 4.12. Statistics

GraphPad Software was used for all the statistical analyses (GraphPad Software for Science Inc., San Diego, CA, USA). To ensure that the data distribution was normal, the values were examined using the Shapiro–Wilk normality test. One-way analysis of variance was used for the statistical analysis. When significance was achieved, the Tukey–Kramer multiple comparison test was employed to compare groups. When the normal distribution of data was not achieved (histology scores), the Kruskal–Wallis test was applied for statistical comparisons. When significance was achieved for Kruskal–Wallis, Dunn’s multiple comparison test was conducted to compare groups. The minimal threshold of significance was set at *p* < 0.05.

## 5. Conclusions

The current work reports that DPG mitigates cadmium-induced testicular damage and impaired spermatogenesis in rats. These promising actions were brought about by the antioxidant, anti-apoptotic, and pro-autophagic effects of DPG, culminating in the improvement of testicular coefficient, serum testosterone levels, and sperm aberrations. Mechanistically, stimulation of the cytoprotective SIRT1/Nrf2/HO-1 axis and the pro-autophagy AMPK/mTOR cascade was proven essential for driving the promising impact of DPG. These findings propose that DPG could be employed as an adjunct approach to mitigate cadmium-induced testicular injury. However, additional investigation is needed to define the detailed molecular pathways of DPG’s action.

## Data Availability

Data are contained within the article.
